# Jasmina Panovska‐Griffiths' discussion contribution to papers in Session 3 of the Royal Statistical Society's special topic meeting on COVID‐19 transmission: 11 June 2021

**DOI:** 10.1111/rssa.12982

**Published:** 2022-12-02

**Authors:** Jasmina Panovska‐Griffiths

**Affiliations:** ^1^ The Big Data Institute and the Pandemic Sciences Institute, Nuffield Department of Medicine University of Oxford Oxford UK; ^2^ The Queen's College University of Oxford Oxford UK

The effective reproduction number *R* has been a headline epidemic metric since the onset of the COVID‐19 pandemic. *R* measures the number of secondary infections arising from an existing infection. At the onset of a new disease, in a fully susceptible population, *R* is the basic reproduction number $R_{0}$ that describes the number of secondary infections stemming from an initial infected case. As the epidemic progresses, *R* reflects the number of secondary infections generated in a population comprising susceptible, exposed, infected, recovered and immune individuals. The growth rate *r*, another widely used epidemic metric throughout the COVID‐19 pandemic, represents the rate at which the epidemic is growing during the exponential phase of epidemic growth. While *R* can be thought as reflective of the level of transmission, *r* can be thought as reflective of the transmission speed.

Since the onset of the SARS‐CoV‐2 epidemic, epidemiological models, calibrated against data on infections, hospital admissions and occupancy and deaths related to COVID‐19, have been widely used to generate outcome metrics such as *R* or *r*. These have been used to inform the status of the epidemic with *R* increasing above 1, and analogously, *r* above 0, suggesting that the epidemic is growing exponentially with the emerging virus spreading fast. Tracking whether *R* and *r* are crossing these thresholds can inform if the epidemic is in a growing or shrinking phase, or the impact of imposed control measures at the time. Generating such metrics across an ensemble of models—which may be different in methodology or on the data they use to fit against—allows a consensus value of *R* and *r* to be derived. The consensus value represents an average outcome across models, and taking a combination of models rather than one model derivative value, allows for uncertainty to be accounted for in the epidemic metrics.

Generating consensus epidemic metrics from models, while useful in informing the epidemic status, has three challenges related to:

Challenge 1: Understanding how to interpret *R* and *r* across different models

Challenge 2: Understanding how *R* and *r* are statistically correlated within and across different models

Challenge 3: Understanding whether *R* and *r* are the most reliable metrics as the epidemic progresses and different interventions are employed

On the first challenge, although *R* and *r* describe broadly similar model outcomes, their exact definition depends on the model structure. For example, in agent‐based models (ABMs) *R* can be directly counted, in population‐based SEIR‐type models *R* is the largest eigenvalue of the generation matrix and hence represents a more abstract concept, while in non‐mechanistic models *R* is typically calculated from the incidence level and the generation time of the circulating variant. In fact within models, *r* and *R* are related via the generation time of the epidemic: the longer the generation time and higher the epidemic growth rate, higher the value of *R*. Appreciating how *R* and *r* are generated and related within models is thus important when we compare these and use them as independent variables within a statistical model used to generate a consensus value.

On the second challenge, we need to be clear on how related are the models and the data being used within models in the ensemble that is generating the consensus value. For example, when combining different models, some of which use different data streams or have different model structures, how does the combined estimate relatively weight their contribution? Or when generating a combined probability distribution, should we use the mean or the median as an average measure and what are the most relevant representative ranges to use: 10th to 90th, 5th or 95th or 25th to 75th percentiles? Finally, when combining model outcomes, what meta‐analysis statistical approach do we use, for example, fixed or random effects models? It is important to be clear whether the generated consensus *R* and *r* are from statistically correlated model outcomes or data—if so the consensus value generated would be strongly weighted to those and statistical methods should be employed to prevent this and get a true representative consensus value.

Finally, on the third challenge, should we in future consider additional metrics, and specifically account for hospitalisation rate in combination to growth rate (via *r*) and transmissibility of the different variants (via *R*)? And separately, should the gold‐standard epidemic metrics be different if we are considering a number of local/regional epidemics that merge to produce a large national epidemic, compared to having a slower growing but geographically large epidemic? And should the consensus epidemic metrics be age‐, settings‐ or variant‐specific?

It is important to address these, and similar challenges in generating epidemic metrics, as we continue utilising mathematical models to study and inform the epidemic status. There is ongoing work in my modelling team that is assessing how to robustly account for uncertainty—in developing models, in setting model parameters and in combining model outcomes—when generating a consensus epidemic metric such as *R*. Learning from the enormous amount of modelling development done at speed during the pandemic, and recognising the aspects that have flourished and can be readily used and those that need to be developed further and adapted to other pathogens, is crucial to be better prepared if another pandemic were to occur.

